# Pavlovian Reward Prediction and Receipt in Schizophrenia: Relationship to Anhedonia

**DOI:** 10.1371/journal.pone.0035622

**Published:** 2012-05-04

**Authors:** Erin C. Dowd, Deanna M. Barch

**Affiliations:** 1 Division of Biology and Biomedical Sciences, Neuroscience Program, Washington University in St. Louis, St. Louis, Missouri, United States of America; 2 Departments of Psychology, Psychiatry, and Radiology, Washington University in St. Louis, St. Louis, Missouri, United States of America; Chiba University Center for Forensic Mental Health, Japan

## Abstract

Reward processing abnormalities have been implicated in the pathophysiology of negative symptoms such as anhedonia and avolition in schizophrenia. However, studies examining neural responses to reward anticipation and receipt have largely relied on instrumental tasks, which may confound reward processing abnormalities with deficits in response selection and execution. 25 chronic, medicated outpatients with schizophrenia and 20 healthy controls underwent functional magnetic resonance imaging using a Pavlovian reward prediction paradigm with no response requirements. Subjects passively viewed cues that predicted subsequent receipt of monetary reward or non-reward, and blood-oxygen-level-dependent signal was measured at the time of cue presentation and receipt. At the group level, neural responses to both reward anticipation and receipt were largely similar between groups. At the time of cue presentation, striatal anticipatory responses did not differ between patients and controls. Right anterior insula demonstrated greater activation for nonreward than reward cues in controls, and for reward than nonreward cues in patients. At the time of receipt, robust responses to receipt of reward vs. nonreward were seen in striatum, midbrain, and frontal cortex in both groups. Furthermore, both groups demonstrated responses to unexpected versus expected outcomes in cortical areas including bilateral dorsolateral prefrontal cortex. Individual difference analyses in patients revealed an association between physical anhedonia and activity in ventral striatum and ventromedial prefrontal cortex during anticipation of reward, in which greater anhedonia severity was associated with reduced activation to money versus no-money cues. In ventromedial prefrontal cortex, this relationship held among both controls and patients, suggesting a relationship between anticipatory activity and anhedonia irrespective of diagnosis. These findings suggest that in the absence of response requirements, brain responses to reward receipt are largely intact in medicated individuals with chronic schizophrenia, while reward anticipation responses in left ventral striatum are reduced in those patients with greater anhedonia severity.

## Introduction

The role of reward processing in the pathophysiology of schizophrenia has garnered significant attention in recent years. Aberrant reward processing has been implicated in both positive [1], [2] and negative [3], [4] symptomatology, and advances in neuroimaging techniques have allowed new insights into the mechanisms of reward processing that may be disrupted in this illness. One such process is reward prediction, or the ability to anticipate a reward when presented with a predictive cue. Reward prediction is strongly associated with dopaminergic activity in the midbrain and striatum [5], [6], which is thought to be dysregulated in schizophrenia [7]. If disruptions in reward signaling prevent predictive stimuli from taking on the appropriate significance, they could contribute to important negative symptoms of schizophrenia such as decreased motivation and anhedonia (a reduced ability to experience pleasure) [3], [4], [8]. Here, we examine this possibility using a Pavlovian reward prediction task to examine functional activity during reward anticipation and receipt in schizophrenia and its relationship to symptoms of anhedonia and amotivation.

A number of previous neuroimaging studies have examined reward prediction in schizophrenia. Studies using monetary incentive delay paradigms have shown reduced ventral striatal responses to reward-predictive cues in patients who are unmedicated [9], or taking typical, but not atypical, antipsychotics [10]. Notably, this reduction in anticipatory activation was associated with negative symptom severity. Several studies in patients taking atypical antipsychotics have shown intact striatal anticipatory activation [11], [12], [13], though some of these studies also demonstrated negative correlations between ventral striatal activation and negative symptoms [12], [13].

Work examining brain responses to reward receipt has also revealed alterations in schizophrenia. Some studies have shown blunted striatal reward responses or prediction errors (responses to outcomes that do not match expectation) [14], [15], [16], [17], [18], while others have shown intact responses [12], [13]. Notably, several of these studies also found inverse relationships between striatal responses to reward receipt and negative or depressive symptoms [12], [13], [14]. In addition, abnormal outcome or prediction error responses have been reported in cortical regions including insula [18] and medial [11], [13], [15], [16], ventrolateral [11], [19], and dorsolateral [13], [20] prefrontal cortex. In several of these studies, attenuated cortical prediction errors or outcome responses were associated with increased severity of either positive [16], [18], [20] or negative [13], [19] symptoms.

Importantly, the literature examining reward processing in schizophrenia has largely relied on instrumental learning tasks, in which rewards must be earned via correct and/or rapid response execution. In these tasks, the ability to anticipate a reward depends upon the ability to earn the reward by responding appropriately. This requires not only reward prediction, but also action selection and response execution, any of which may be impaired in schizophrenia. Here, we examined reward prediction in schizophrenia in the absence of requirements for response selection and execution. Using a passive Pavlovian paradigm, we examined functional activation in response to rewarding stimuli and to predictive cues that had been associated with rewards based on pre-scan instructions.

Previous work using aversive Pavlovian conditioning has revealed abnormal brain responses among individuals with schizophrenia. Using a task in which colored cues were associated with affectively negative or neutral pictures, Romaniuk et al [21] demonstrated reduced responses to aversive cues in bilateral amygdala, as well as decreased prediction error responses in the midbrain. Further, inappropriate midbrain activation to neutral stimuli correlated with delusional symptom severity. Similarly, work by Jensen et al [22] using aversive noise stimuli revealed increased right ventral striatum activation to neutral cues in patients. These studies suggest that even in the absence of response requirements, brain responses to neutral cues in an aversive context may be augmented among individuals with schizophrenia. In addition, two studies have examined functional activity using Pavlovian paradigms with appetitive rewards in schizophrenia [14], [23]. Waltz et al used a timing-sensitive paradigm to examine anticipation and receipt of primary reward (juice), and found reduced positive (but not negative) prediction errors and reward responses in schizophrenia in widespread regions throughout the brain. However, integrating these results with others in the literature is challenging because the timing-sensitive paradigm and primary reward differ greatly from the tasks typically used in instrumental studies. Morris et al used a Pavlovian prediction error task to examine responses to expected and unexpected rewards and omissions. In this study, ventral striatal responses in patients were intact for reward receipt vs. omission, but failed to differentiate between expected and unexpected rewards. However, this study did not examine brain activity at the time of cue presentation. Here, we used a Pavlovian monetary reward prediction task to examine whether functional activation during reward anticipation and receipt is altered in schizophrenia even in the absence of response requirements, and whether it relates to symptoms of anhedonia and amotivation.

## Materials and Methods

### Participants

Participants were 29 stable outpatients with DSM-IV schizophrenia or schizoaffective disorder and 22 healthy controls with no personal or family history of psychosis. All patients were taking antipsychotic medications, which were stable for at least two weeks. Participants were group matched on sex, age, parental education, handedness [24], and smoking status. Inclusion criteria were 1) age 18–50 years and 2) ability to give informed consent. Exclusion criteria were 1) DSM-IV substance abuse or dependence within the past 6 months (except nicotine); 2) DSM-IV major depressive disorder or dysthymia in the past year; 3) past head injury with neurological sequelae and/or loss of consciousness; 4) DSM-IV mental retardation, and 5) any contraindication to MRI including pregnancy, claustrophobia, any metallic object in the body, etc. Participant diagnoses were based on a Structured Clinical Interview for DSM-IV-TR [25] conducted by a Masters-level clinician. Clinical symptoms were rated using the Scales for the Assessment of Positive Symptoms (SAPS) [26] and Negative Symptoms (SANS) [27], and summarized using the following symptom domain scores [28]: 1) positive symptoms – hallucinations and delusions; 2) negative symptoms – alogia, anhedonia, avolition, affective flattening and attentional impairment; and 3) disorganization – bizarre behavior, positive thought disorder, and inappropriate affect. Anhedonia was assessed using the Chapman revised physical and social anhedonia scales [29], [30], [31]. This study was conducted in accord with APA standards for ethical treatment of human subjects. Written informed consent was obtained from all participants, and all procedures were approved by the Washington University Human Research Protection Office. Participant demographic and clinical characteristics are shown in Table 1.

**Table 1:** Clinical and demographic characteristics.

	CON	SCZ	
Age	33.20 (9.44)	31.44 (9.31)	
Education (years)	15.03 (2.34)	12.60 (2.40)*	
Highest Parental Education (years)	14.00 (1.95)	13.96 (3.08)	
Sex (% Male)	70	72	
Race (% Caucasian)	60	52	
Smoking status (% Smokers)	25	40	
Past Major Depressive Disorder (%)	5	12	
Past Substance Dependence (%)	10	24	
Chapman Social Anhedonia	2.00 (1.61)	3.50 (2.50)*	
Chapman Physical Anhedonia	2.44 (1.76)	6.00 (3.35)*	
Duration of Illness (years)	-	13.93 (8.36)	
**Antipsychotic Medication**		**% Taking**	**Average Dose (mg)**
Fluphenazine decanoate	-	4.0	25.00
Haloperidal		4.0	10.00
Haloperidal decanoate	-	12.0	53.33
Risperidone		24.0	3.58
Aripiprazole	-	24.0	26.00
Paliperidone	-	12.0	8.00
Clozapine	-	8.0	250.00
Olanzapine	-	4.0	20.00
Quetiapine	-	24.0	266.67
Ziprasidone	-	8.0	125.00
**Other Medication**			
Antidepressant	-	28.0	
Mood Stabilizer	-	16.0	
Anticholinergic	-	28.0	
SAPS/SANS Positive	-	2.10 (2.40)	
SAPS/SANS Negative	-	2.95 (3.64)	
SAPS/SANS Disorganization	-	0.72 (0.77)	

* p<.05; CON = control, SCZ = schizophrenia, SD = standard deviation, SAPS/SANS = Scale for the Assessment of Positive/Negative Symptoms (Andreasen 1983).

### Materials and Tasks

All participants underwent fMRI while performing a Pavlovian reward prediction task. Subjects were presented with one of two visual cues (pink cross or green circle), one predicting receipt of 75¢ for the trial, and one predicting receipt of 0¢ for the trial. The cues were followed by their predicted outcome 75% of the time, and with the opposite outcome 25% of the time. Participants were informed of the cue-outcome associations before the scan, and were told that the cue usually, but not always, predicted its associated outcome. They were also told that they could keep any money they were awarded during the task. In each trial, the visual cue was presented for 10 seconds, followed by a symbol indicating the outcome (+75¢ or +0¢) for 4 seconds. Inter-trial intervals varied pseudorandomly between 4 and 8 seconds. Participants completed four runs of 16 trials each, for a total of 64 trials (32 per cue type). Participants were paid $25/hour for their time, and were awarded an additional $20 in reward money upon session completion.

### Image Acquisition and Processing

Imaging data was acquired on a 3T Siemens TIM TRIO system with a 12-channel head coil. High-resolution T1 images (TE = 3.16ms, TR = 2400ms, 176 slices, 1X1X1mm voxels) and T2 images (TE = 96 ms, TR = 5s, 48 slices, 1.02×1×3 mm voxels) were acquired to aid in registration to a common atlas space. Functional images were collected in four runs of 182 frames each using an asymmetric spin-echo echo-planar sequence (TR = 2000 ms, TE = 27 ms, FOV = 256 mm, flip = 90°, 33 slices). Functional runs acquired axial images parallel to the anterior-posterior commissure plane with 4 mm^3^ isotopic voxels. Stimuli were presented using PsyScope on a G3 Macintosh, with each trial onset triggered directly by a pulse from the scanner. The MR data was normalized across runs by scaling whole-brain signal intensity to a fixed value and removing the linear slope on a voxel-by-voxel basis to counteract effects of drift [32]. The MR data was then aligned to correct for head motion using rigid-body rotation and translation correction algorithms [33], [34], [35], which provide estimated movement parameters used to evaluate movement differences between groups. We also compared signal-to-noise ratios (SNR = mean/variance) between groups [36], and removed runs or participants with movement or SNR values not meeting predetermined criteria (Text S1 and Table S1). Of the 29 patients and 22 controls who underwent the experimental protocol, four patients and two controls were excluded for excessive head motion, yielding the final sample of 20 controls and 25 patients. The images were then resampled into 3 mm^3^ voxels, registered to Talairach space using 12-parameter affine transformations, and smoothed with a 6 mm FWHM Gaussian filter.

### fMRI Data Analysis

All functional data was analyzed using in-house software. Data analysis was conducted using general linear models (GLMs) [33], [37], [38], which included task-related regressors as well as nuisance regressors for linear trends within runs and baseline shifts between runs. Canonical hemodynamic response shapes (Boynton functions) were used to estimate cue- and receipt-related activation. Regressors included two cue types (money and no-money) and four outcome types (expected money, expected no-money, unexpected money, unexpected no-money). The parameter estimates from the GLMs for each subject were entered into ANOVAs using subject as a random factor. To identify regions in which activation related to anticipation of reward (cue-related activity), we performed a repeated measures ANOVA with cue type (money, no-money) as a within-subjects factor and group (schizophrenia, control) as a between subjects factor. To identify regions in which activation related to reward receipt, we used cue type (cue money, cue no-money) and receipt type (receive money, receive no-money) as within-subjects factors and group (schizophrenia, control) as a between-subjects factor. Cue type was included as a factor in analyses of receipt in order to evaluate potential prediction error effects, which would be expected to modulate responses to receipt according to whether the outcome was expected or unexpected.

These ANOVAs were used in voxelwise whole-brain and ROI analyses. Whole-brain analyses were corrected for multiple comparisons using a p-value/cluster size threshold of p<.003 (two-tailed) and 13 voxels. This correction factor was determined by Monte Carlo simulations to provide a whole-brain false-positive rate of p<.05 [39], [40]; an approach equivalent to that employed by the Alphasim program in the AFNI software package. Second, voxelwise ROI analyses were conducted within an a priori mask consisting of a network of regions implicated in reward processing, an approach equivalent to the “small volume correction” procedure in the SPM software package. This mask, developed by Beck et al [41], consisted of regions that were hand-drawn in Talairach space on the basis of anatomical landmarks and previously published coordinates [42], [43], [44], [45], [46], [47], [48], [49], [50], including the dorsal and ventral striatum, ventral tegmental area, substantia nigra, amygdala, orbitofrontal cortex (OFC), ventromedial prefrontal cortex (VMPFC), and insula (Figure S1). This analysis was corrected for multiple comparisons using a combined p-value/cluster size threshold (p<.01 (two-tailed) and 19 voxels) determined using Monte Carlo simulations to provide α<.05 for the whole ROI mask.

We also conducted correlation analyses between BOLD contrasts and anhedonia scores. In these analyses, Cue Money – No-money and Receive Money – No-money contrasts were created for each subject and correlated with Chapman physical and social anhedonia scores. These correlations were conducted voxelwise, and were corrected using the same small-volume and whole-brain correction procedures described above. To explore whether these relationships were unique to the patient group, regions demonstrating significant correlations were also examined within the control group.

## Results

### Participant Characteristics

Participant demographic and clinical characteristics are presented in Table 1. The patient and control groups did not differ significantly on age, sex, race, smoking status, or parental education. The control group demonstrated significantly higher personal education than the patient group. With respect to anhedonia severity, individuals with schizophrenia demonstrated significantly higher scores than controls on both the Chapman physical (t(38) = –4.06, p<.001) and social (t(38) = –2.20, p<.001) anhedonia scales.

### fMRI Results: Reward Anticipation

Results of the voxelwise ROI and whole-brain analyses are reported in Table 2. No regions demonstrated a significant main effect of cue. There was a significant main effect of group in VMPFC in the ROI analysis and significant main effects of group in VMPFC, cerebellum and left posterior cingulate in the whole-brain analysis, all of which demonstrated greater activation overall in controls than in patients. In addition, a significant Cue X Group interaction was seen in a region in right anterior insula in the ROI analysis. This region showed greater activity for no-money than money cues in controls (F(1,19) = 8.74, p<.009), and greater activity for money than no-money cues in patients (F(1,24) = 8.15, p<.009). There were no significant Cue X Group interactions in the whole-brain analysis.

**Table 2:** Cue-related activation.

Effect	Analysis	Brain Region	Brodmann Area	Talairach Coordinates	Voxels	Z	Activation Pattern
							
Group	ROI	VMPFC	32/10	+0, +44, +8	93	3.63	CON > SCZ
	WB	R Cerebellum	-	+14, –57, –46	18	4.06	CON > SCZ
		R VMPFC	32/10	+1,+47,+8	58	3.95	CON > SCZ
		L VMPFC	32/10	–12, +44, +0	20	3.75	CON > SCZ
		L Posterior Cingulate	31	–2, –38, +34	23	3.74	CON > SCZ
CueXGroup	ROI	R Anterior Insula	13	+32, +15, +0	25	3.79	CON: No Money > Money; SCZ: Money > No Money

R = Right, L = Left, CON = control, SCZ = schizophrenia, VMPFC = Ventromedial prefrontal cortex. Z values represent mean activation across the region.

**Figure 1:**
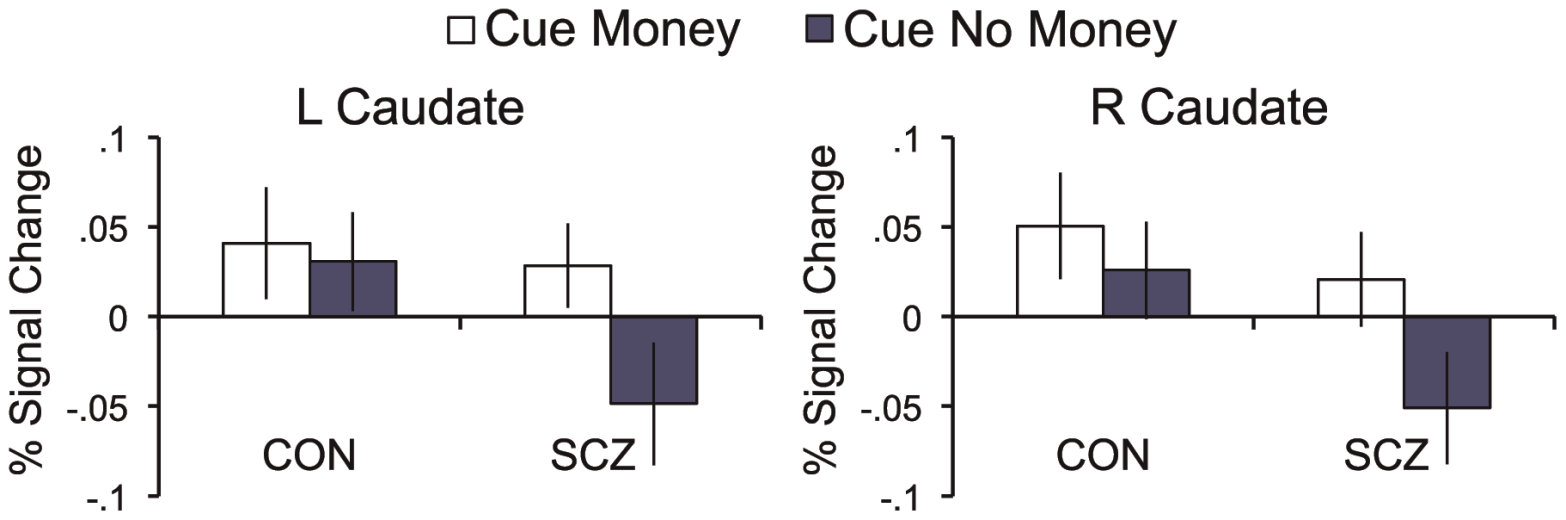
Cue-related activation in bilateral caudate ROIs. Activation shown is mean activation across voxels within regions of interest. Error bars represent standard error.

Given past findings with similar paradigms, we had expected to see greater activity for money than no-money cues in striatal regions among controls. Thus, to further explore the nature of the striatal responses within each group, we extracted the mean activation across voxels within caudate, putamen, and nucleus accumbens ROIs ([51]; Figure S2) for money and no-money cues. Cue (Money, No-Money) X Group (Control, Schizophrenia) ANOVAs revealed a significant main effect of cue within bilateral caudate (left: F(1,43) = 4.16, p<.05; right: F(1,43) = 5.12, p<.03), with greater activation to money than no-money cues (Figure 1). Post-hoc paired t-tests revealed that this effect was driven largely by the patient group, which demonstrated significant activation to money cues and deactivation to no-money cues (left: t(24) = 2.33, p<.03; right: t(24) = 2.23, p<.04). Controls showed activation to both money and no-money cues, with no significant differences between cue types. No other regions demonstrated a main effect of cue, and there were no significant main effects of group or Cue X Group interactions in the striatal ROIs. We also wished to examine the possibility that stronger anticipatory responses were evident only in the later runs, which could have resulted if the stimulus-outcome associations that were formed based on pre-scan instruction were strengthened by experience during the early trials of the scan. To this end, we repeated our cue-related analyses after excluding the first BOLD run, in essence treating this run as a practice. This analysis did not reveal any additional reward-related regions showing significant main effects of cue or cue X group interactions.

### fMRI Results: Reward Receipt

**Figure 2:**
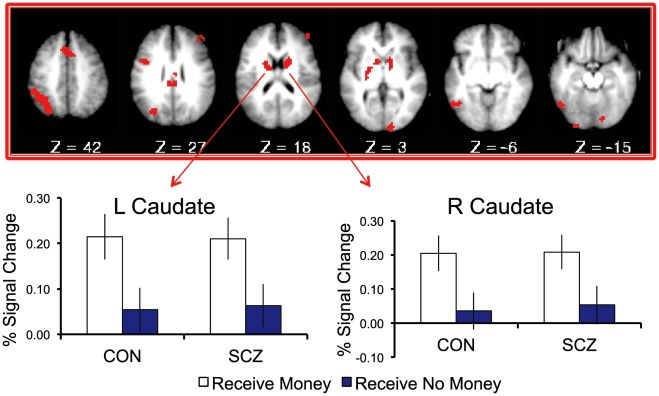
Regions demonstrating a significant main effect of receipt. All regions showed greater activation for receipt of money than no money. Both ROI results (threshold of p<.01 and 19 voxels within ROI mask) and whole-brain results (threshold of p<.003 and 13 voxels) are displayed. Graphs represent mean activation magnitudes across voxels within example regions among individuals with schizophrenia (SCZ) and controls (CON). Error bars represent standard error.

**Figure 3:**
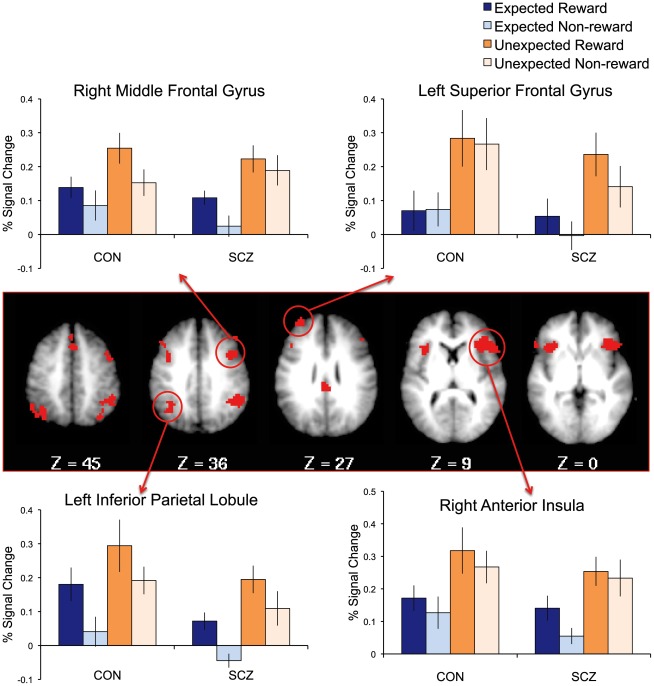
Regions demonstrating a Cue X Receipt interaction. All regions showed greater activity for unexpected than for expected outcomes. Both ROI results (threshold of p<.01 and 19 voxels within ROI mask) and whole-brain results (threshold of p<.003 and 13 voxels) are displayed. Graphs represent mean activation magnitudes across voxels within example regions among individuals with schizophrenia (SCZ) and controls (CON). Error bars represent standard error.

Results of voxelwise ANOVA analyses are presented in Table 3. The ROI analysis revealed a main effect of receipt in bilateral caudate, which showed greater activation for receipt of money than no-money. On whole brain analysis, a number of additional regions also showed this pattern, including bilateral DLPFC, dorsomedial PFC, left posterior parietal cortex, and several occipital and cerebellar regions (Figure 2). Post-hoc tests revealed that each group individually demonstrated greater activation for money than no-money receipt within each region at trend level or higher. In addition, several regions demonstrated a significant Cue X Receipt interaction (Figure 3). These included bilateral anterior insula and left ventrolateral prefrontal cortex, which activated more strongly for unexpected than expected outcomes (i.e. when money was cued but no-money received, or no-money cued and money received). Additional regions showing this pattern were also identified on whole-brain analysis. These included several regions commonly implicated in cognitive control and working memory [52], [53], such as bilateral DLPFC, DMPFC, posterior parietal cortex, and anterior insula/frontal operculum. Post-hoc tests revealed that each of these regions demonstrated a significant cue X receipt interaction within each group separately (Table 3). To further explore the patterns of activity in these regions, we conducted paired t-tests comparing unexpected versus expected rewards and unexpected versus expected non-rewards within each group (Table S2). All regions demonstrated significantly greater activation for unexpected than expected outcomes when collapsing across reward versus nonreward. When reward and non-reward were examined separately, all regions demonstrated greater activation for unexpected than expected rewards and for unexpected than expected non-rewards, though not every effect was significant in each group (details presented in Table S2). For example, the region in left ventrolateral prefrontal cortex showed significantly greater activation for unexpected than expected rewards in controls, but not patients, and for unexpected than expected non-rewards in patients, but not controls (Figure S3). These results suggest that the activation patterns driving the cue X receipt interactions were not identical between groups. However, no interactions with group were present, and both groups did show clear effects of unexpected versus expected outcomes. In addition to the regions showing main effects of receipt or cue X receipt interactions, a main effect of group was seen in right DLPFC, which demonstrated greater activity in controls than patients, as well as in left pre- and post-central gyri, which activated more strongly in patients than controls. Finally, one region in right inferior temporal gyrus demonstrated a Receipt X Group interaction wherein controls responded more strongly to no-money than money receipt, while patients responded more strongly to money than no-money receipt.

**Table 3:** Receipt-related activation.

								Post-hoc Tests:	
Effect	Analysis	Brain Region	Brodmann Area	Talairach Coordinates	Voxels	Z	Activation Pattern	CON	SCZ
Receipt	ROI	L Caudate	-	–15, –4, +10	99	3.92	Money > No Money	p<.002	p<.02
		R Caudate	-	+12, +2, +13	74	3.94	Money > No Money	p<.001	p<.02
	WB	R Cerebellum	-	+1, –28, –41	40	4.09	Money > No Money	p<.02	p<.002
		L Cerebellum	-	–10, –46, –40	70	4.50	Money > No Money	p<.003	p<.002
		R Cerebellum	-	+11, –40, –43	15	3.70	Money > No Money	p<.03	p<.004
		L Cerebellum	-	–35, –62, –24	47	4.48	Money > No Money	p<.07	p<.001
		R Cerebellum	-	+53, –63, –24	15	4.38	Money > No Money	p<.005	p<.001
		R Cerebellum	-	+35, –65, –23	39	4.60	Money > No Money	p<.03	p<.001
		R Cerebellum	-	+12, –79, –19	22	4.14	Money > No Money	p<.03	p<.001
		L Middle Occipital Gyrus	37	–47, –62, –8	75	4.55	Money > No Money	p<.02	p<.001
		L Inferior Occipital Gyrus	18	–37, –84, –13	18	3.84	Money > No Money	p<.03	p<.002
		L Fusiform Gyrus	18	–25, –92, –12	20	4.12	Money > No Money	p<.02	p<.002
		R Cuneus	17	+11, –96, +1	40	3.86	Money > No Money	p<.06	p<.001
		L Thalamus	-	–12, –7, +10	107	4.39	Money > No Money	p<.001	p<.004
		R Caudate	-	+13, +1, +11	92	4.38	Money > No Money	p<.001	p<.005
		R Middle Frontal Gyrus	46	+43, +40, +22	44	4.18	Money > No Money	p<.002	p<.005
		L Inferior Frontal Gyrus	9	–42, +4, +26	55	3.61	Money > No Money	p<.03	p<.005
		Cingulate Gyrus	23	+0, –27, +28	65	4.14	Money > No Money	p<.006	p<.003
		L Angular Gyrus	39	–37, –57, +38	359	4.36	Money > No Money	p<.002	p<.003
		L Precuneus	7	–12, –74, +34	22	3.77	Money > No Money	p<.07	p<.002
		L Superior Frontal Gyrus	6	–2, +9, +48	141	4.07	Money > No Money	p<.02	p<.001
		L Precentral Gyrus	4	–44, –13, +42	16	3.59	Money > No Money	p<.1	p<.001
Group	WB	R Middle Frontal Gyrus	9	+43, +29, +33	14	3.76	CON > SCZ	-	-
		L Precentral Gyrus	4	–34, –28, +64	17	3.47	SCZ > CON	-	-
		L Postcentral Gyrus	3	–10, –32, +67	15	3.87	SCZ > CON	-	-
Cue X Receipt	ROI	L Ventrolateral PFC	47	–44, +22, –6	20	3.41	Unexpected > Expected	p<.001	p<.04
		R Anterior Insula	13	+34, +17, +4	57	4.12	Unexpected > Expected	p<.002	p<.005
		L Anterior Insula	13	–34, +16, +4	60	3.68	Unexpected > Expected	p<.005	p<.02
	WB	R Anterior Insula	13/45	+41, +21, +4	208	4.55	Unexpected > Expected	p<.002	p<.002
		L Anterior Insula	13/45	–40, +17, +0	89	4.21	Unexpected > Expected	p<.001	p<.009
		L Superior frontal Gyrus	9	-31, +47, +28	26	4.12	Unexpected > Expected	p<.006	p<.003
		L Posterior Cingulate	43	–1, –31, +26	25	3.39	Unexpected > Expected	p<.008	p<.04
		R Middle Frontal Gyrus	6	+42, +11, +42	137	4.65	Unexpected > Expected	p<.02	p<.001
		L Middle Frontal Gyrus	9	-44, +19, +32	18	3.84	Unexpected > Expected	p<.02	p<.004
		L Middle Frontal Gyrus	6	–39, +4, +34	14	3.65	Unexpected > Expected	p<.003	p<.02
		R Inferior Parietal Lobule	40	+44, –52, +40	151	4.40	Unexpected > Expected	p<.04	p<.001
		L Inferior Parietal Lobule	7	–39, –60, +42	139	4.11	Unexpected > Expected	p<.03	p<.001
		L Superior Frontal Gyrus	8	–1, +20, +48	67	4.17	Unexpected > Expected	p<.002	p<.007
Receipt X Group	WB	R Inferior Temporal Gyrus	37	+51, –70, +3	17	3.56	CON: No Money > Money SCZ: Money > No Money	-	-

R = Right, L = Left, ROI = Region of Interest analysis, WB = Whole Brain analysis, CON = control, SCZ = schizophrenia, PFC = prefrontal cortex. Z and p values represent mean activation across the region.

### Correlations with Anhedonia

#### Cue-related activity correlations

**Figure 4:**
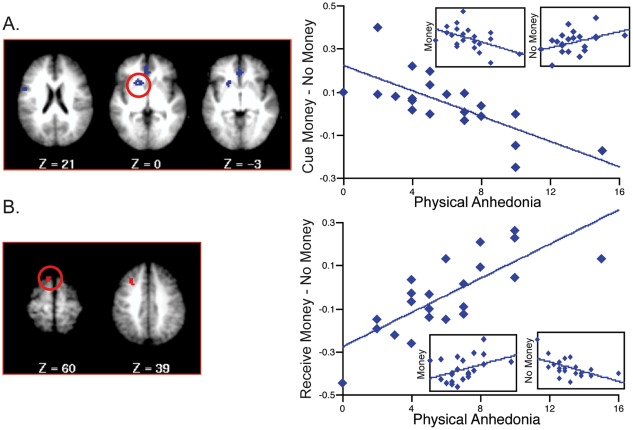
Regions demonstrating correlations between activation and anhedonia severity. (A) Results of voxelwise correlation between Chapman physical anhedonia score and the Cue Money – No Money contrast in patients, ROI analysis (threshold of p<.01 and 19 voxels within ROI mask). (B) Results of voxelwise correlation between Chapman physical anhedonia and the Receive Money – No Money contrast in patients, whole brain analysis (threshold of p<.003 and 13 voxels.) Graphs represent mean activation across the region.

In the patient group significant negative correlations were found between Chapman physical anhedonia and the Cue Money – No-money contrast in regions in left ventral striatum and VMPFC (Figure 4A). As shown in Table 4, post-hoc analyses revealed that this relationship was primarily driven by activation in response to money cues, which correlated negatively with physical anhedonia, rather than by activation in response to no-money cues. This indicates that in patients, greater physical anhedonia is associated with less ventral striatal activity during the anticipation of rewards. On whole-brain analysis, a similar pattern was observed in left inferior frontal gyrus. In VMPFC, the negative correlation was driven by activation to both the money cue, which correlated negatively with physical anhedonia, and the no-money cue, which correlated positively with physical anhedonia. Thus, individuals who were higher in anhedonia demonstrated both decreased responses to money cues and increased responses to no-money cues in VMPFC. To determine whether these relationships were also present among controls, we conducted correlation analyses in the control group between the Cue Money – No Money contrast and physical anhedonia scores within the ventral striatal and VMPFC regions that had reached significance in the patient group (Table 4). A significant negative correlation was found within the VMPFC region, indicating that greater physical anhedonia is associated with lower VMPFC anticipatory activity among both patients and controls. In the ventral striatal region, there was a negative relationship between anhedonia and cue-related activity in controls, but this relationship failed to reach significance. No significant regions were identified for social anhedonia.

**Table 4:** Correlations between Cue- and Outcome-Related Activity and Anhedonia Scores.

Correlation		Analysis	Brain Region	Brodmann Area	Talairach Coordinates	Voxels	r	Money r	No-Money r	CON r
Cue Money - No Money	Physical Anhedonia	ROI	L Ventral Striatum	-	–17, 11, –1	27	−.720*	−.482*	.408	–.303
		ROI	L VMPFC	32	–3, 40, 4	20	−.652*	−.426*	.643*	−.493*
		WB	L VMPFC	24	–2, 33, 0	37	−.726*	−.502*	.670*	–.459
		WB	L Inferior Frontal Gyrus	44	–51, 1, 20	14	−.726*	−.736*	.148	.100
Receive Money - No Money	Physical Anhedonia	WB	L Middle Frontal Gyrus	8	–30, 20, 38	23	.722*	.351	−.457*	–.186
		WB	L Superior Frontal Gyrus	6	–11, 24, 58	14	.762*	.403	−.553*	−.627*
Receive Money - No Money	Social Anhedonia	WB	L Uncus	20	–15, –7, –34	31	−.772*	−.649*	.619*	–.026
		WB	R Cerebellum	-	1, –47, -27	18	−.738*	−.569*	.477*	.337

* p<.05. R = Right, L = Left, ROI = Region of Interest analysis, WB = Whole Brain analysis, CON = Control, SCZ = Schizophrenia. r values represent mean activation across the region.

#### Receipt related activity correlations

At the time of receipt, significant positive correlations were seen between physical anhedonia and the Receive Money – No-money contrast in left superior and middle frontal gyri (Figure 4B). These correlations were driven primarily by responses to no-money receipt, which correlated negatively with physical anhedonia. In other words, patients who were higher in anhedonia showed less activation to no-money receipt in these regions. In addition, social anhedonia correlated negatively with activity in left uncus and right cerebellum in the Receive Money – No-money contrast. These relationships were driven by activity in response to the receipt of both money and no-money; thus, patients with higher social anhedonia showed smaller responses in these regions to money receipt and larger responses to no-money receipt. In controls, only the left superior frontal gyrus showed a significant correlation, which was in the negative direction.

### Medication Analyses

To explore the possibility that antipsychotic medications may have influenced the results, we conducted correlations between medication dose in chlorpromazine equivalents [54]and cue- or receipt-related brain activation. First, we examined the regions that had shown correlations between cue- or receipt-related activity and anhedonia ratings, none of which showed significant correlations between antipsychotic medications and the relevant contrast (all *p* values >0.16). Similarly, antipsychotic dosage failed to correlate significantly with physical or social anhedonia scores (all *p* values >0.4). To look for more general medication effects, we also conducted voxelwise ROI and whole-brain correlations between antipsychotic dose and activation for the money – no-money cue and receipt contrasts. There were no regions whose cue-related activity correlated significantly with antipsychotic dosage. For receipt, no regions within the ROI mask correlated significantly with medication dose. Whole-brain analysis revealed two regions that correlated positively with dose, such that higher doses were associated with greater responses to receipt of money versus no-money. One of these regions was within left prefrontal cortex (–30, 55, 0; 14 voxels) and the other fell within the lateral ventricles adjacent to caudate (3, 11, 12; 22 voxels).

In addition, given past results showing effects of typical vs. atypical antipsychotic medications on striatal anticipatory activation, we wished to determine to what extent our results were affected by medication type. To do this, we repeated our analyses with the 5 subjects taking typical antipsychotics removed (unfortunately, we had too few subjects taking typical antipsychotics to examine this group separately). All ANOVA and correlation results reported in Tables 2, 3, and 4 remained significant at trend level or above, though some decreased slightly in significance perhaps reflecting the reduction in power. Further, no new correlations or group effects on striatal activation emerged at the time of cue presentation or receipt.

## Discussion

The goal of this study was to examine cue- and receipt- related brain activation during Pavlovian reward prediction in schizophrenia, and to examine the relationship between this activation and symptoms of anhedonia and amotivation. Our results demonstrate few activation differences at the group level during reward anticipation and receipt. However, we observed that left ventral striatal and VMPFC activation during reward anticipation in schizophrenia was reduced in patients who are higher in anhedonia. These findings are consistent with a number of studies showing similar results in instrumental paradigms, consistent with the interpretation of these studies as reflecting abnormal reward prediction mechanisms in patients experiencing anhedonia and/or motivational deficits.

### Cue-related Activation

At the group level, we did not see striatal cue-related activity that differed significantly between groups. Upon ROI analysis, there was a main effect of cue in bilateral caudate with greater activity for money than no-money cues, an effect that was driven by the patient group and was not significant in controls alone. We speculate that this relative lack of differential cue-related activity among controls as compared to published studies may reflect a difference between our passive Pavlovian paradigm and the instrumental paradigms typically used in reward prediction studies. Instrumental tasks require that information in the cue be used to plan and execute a correct motor response, which may enhance cue-related activation as compared to Pavlovian paradigms. Our task, modeled after O’Doherty et al 2002 [55], was designed to be completely passive in order to eliminate confounding variables associated with execution of a motor response. However, the O’Doherty study used primary reward (juice), and it is possible that the monetary rewards used here were less salient and therefore less capable of eliciting the expected cue-related activation. Similarly, it is possible that the monetary rewards were more salient for patients than controls given their lower socioeconomic status, which may underlie the finding of significant striatal anticipatory activation in patients but not controls.

Outside the striatum, we saw group differences in cue-related activity in the right anterior insula, such that patients showed greater activation to money than no-money cues, while controls showed the opposite pattern. The insula has been implicated in the detection of, and allocation of attention to, salient events [56], and has been shown to respond to unpleasant stimuli, including cues predicting negative [50] or low-expected-value [57] outcomes. This is consistent with the pattern seen in controls, where insula activation was strongest to the less-pleasant, less-frequent cue. Patients, on the other hand, showed the opposite pattern, consistent with prior hypotheses [1] about an altered pattern of salience attribution in this group.

### Receipt-related Activation

Patients and controls showed similar activation at the time of reward receipt. A number of regions including bilateral caudate responded more strongly to money than no-money receipt in both groups, suggesting that brain responses to reward receipt were largely intact in this sample of patients. Similarly, we found greater responses to unexpected than expected outcomes in both groups in several regions of the cognitive control network [58], such as bilateral DLPFC, anterior insula, and posterior parietal cortex. These findings suggest that expectancy violations resulted in the recruitment of cognitive control regions in both patients and controls.

The results reported here are compatible with some, but not all, aspects of the results reported by Morris et al [23]. Both studies showed globally intact responses to reward receipt vs. omission, with both patients and controls showing reward responses in regions such as midbrain, insula, cingulate, and inferior frontal cortex. However, unlike Morris et al, we showed surprise responses in a number of cortical regions in both groups. In addition, while Morris et al reported group differences in ventral striatal prediction error activity, we failed to identify any regions whose activity pattern resembled a prediction error signal. This result is not surprising given the design of the task, which was intended to examine reward anticipation and receipt, not prediction error signaling. Because appreciable learning was not expected to occur and cue-outcome contingencies remained constant during the session, one would expect prediction error activity to be minimal.

Overall, our results demonstrate a pattern of largely intact responses to reward receipt throughout the brain at the group level among individuals with schizophrenia. The robust striatal responses to reward receipt among individuals with schizophrenia are consistent with recent studies demonstrating intact outcome responses in this region [12], [13]. However, the pattern of intact cortical responses to reward receipt seen here contrasts with recent findings [11], [13], [19]. One possible source of this discrepancy is that the referenced studies used instrumental tasks, which may engage cortical structures upon reward receipt to a greater extent than Pavlovian paradigms due to the requirements for updating future action plans. These results therefore suggest that responses to receipt of monetary reward per se may be intact in schizophrenia, and that reward processing deficits in this illness may therefore lie downstream in processes required to translate reward information into action plans. This conclusion is consistent with a large body of data suggesting intact hedonic responses to pleasant stimuli in schizophrenia, despite clinically evident deficits in motivation [59]. It is also consistent with the view that motivational deficits in schizophrenia are related to deficits in value representation [3]. In order to influence goal-directed behavior, information about reward receipt must be integrated and represented in a way that makes it available to guide value-based decision-making. Deficits in this process may not be evident during simple Pavlovian tasks, but may become evident in tasks where reward information must be used to guide future choices.

### Relationships to Anhedonia

Correlation analyses revealed an inverse relationship between anticipatory activity in left ventral striatum and VMPFC and Chapman physical anhedonia scores in the patient group. This relationship is consistent with the findings of several reward prediction studies in schizophrenia showing that higher negative symptoms, particularly anhedonia and avolition, are associated with reduced striatal responses to reward-predicting cues [9], [12]. Along with Waltz et al [14], we have shown that this relationship is present in Pavlovian paradigms, suggesting that patients who are higher in anhedonia/avolition have larger deficits in reward prediction processes even in the absence of response requirements. Interestingly, the negative correlation seen in patients within the VMPFC region was also significant among controls, suggesting a relationship between activation to reward-predictive cues and individual differences in anhedonia irrespective of diagnosis. Previous studies in the literature examining non-clinical samples have identified relationships between anhedonia severity and brain activity in similar regions. Harvey et al [60] showed a negative correlation between anhedonia severity and activation in a rostral anterior cingulate cortex (rACC) region that overlaps with the VMPFC region identified here, and EEG studies by Wacker et al [61] showed increased resting delta activity (i.e. decreased resting activity) in rACC among more severely anhedonic individuals. These findings suggest that altered VMPFC activity is associated with anhedonia as a clinical dimension that is elevated in and associated with vulnerability to schizophrenia [62], [63], but which crosses diagnostic boundaries.

We also observed relationships between anhedonia scores and activation at the time of receipt. In left frontal cortex, higher physical anhedonia was associated with decreased responses to nonrewarding outcomes. Because this study did not have a punishment condition and nonreward may have been considered a negative outcome in this context, this relationship may perhaps be thought of as a blunted response to negative outcomes among the more severely anhedonic patients. This finding is similar to past results, where anhedonia was found to relate to a reduced experience of both positive and of negative emotion [64].

When considering the relationships between anhedonia and brain activity in this study, it is worth noting that our sample had lower mean anhedonia scores than previous studies in the literature [11], [64]. Given the relationship found between physical anhedonia and anticipatory activity in patients, it is possible that a sample of patients who were higher in anhedonia may have shown differences at the group level between patients and controls, and that differences in anhedonia severity between samples may underlie some of the discrepant findings in the literature.

There is ample evidence from basic science research that hedonic experience and motivation are dissociable constructs subserved by different neurobiological systems [65]. However, the majority of clinical and self-report instruments available to assess anhedonia in schizophrenia, including the Chapman physical and social anhedonia scales used here, predate these findings and tend to contain items relevant to both constructs [66]. Here, we used the Chapman scales because of their wide range, which is well-suited to correlation analyses, as well as their widespread use and demonstrated reliability in the schizophrenia literature. It is important to note, however, that the non-specificity of these scales means that the relationships seen here may be driven by deficits in motivated behavior rather than by deficits in the experience of pleasure per se, consistent with the idea that a reduced ability to anticipate rewards may contribute to deficits in goal-directed behavior. Future studies using new clinical instruments that carefully distinguish between hedonic and motivational deficits, such as the Clinical Assessment Interview for Negative Symptoms scale [67], will be required to dissociate the relationships of these two constructs to reward processing in schizophrenia.

### Limitations

The passive task used in this study, while essential to address our experimental question about reward processing in the absence of response requirements, also confers an important limitation in that it did not allow the collection of behavioral data demonstrating attention to the task. This raises the possibility that our failure to find striatal reward anticipation responses among controls may have been driven by lack of attention to the stimuli. However, while we did not see significant *differential* responses between Money and No-money cues, we did see robust responses to cue presentation (irrespective of cue type) in regions including visual cortex, anterior insula, DLPFC, and posterior cingulate (Figure S4). Further, we saw clear effects of money vs. no-money receipt in several brain regions within both groups, indicating that participants were attending to the outcome stimuli. Finally, we saw differential responses to unexpected versus expected outcomes, suggesting that participants were attending to the cues, developing cue-based expectancies, and reacting to expectancy violations upon outcome presentation. Together, we feel that these results provide reasonable evidence that participants were indeed attending to the stimuli. We acknowledge that the degree of attentional engagement here was likely less than that required by active tasks, which is an additional reason to speculate that the relative lack of differential cue-related activity may reflect a difference between the Pavlovian paradigm used here and instrumental paradigms in the literature. An additional consequence of the passive task is that no behavioral measure of learning was available to determine whether stimulus-outcome associations were fully formed based on pre-scan instructions, or whether they were strengthened by experience during the early trials of the scan. Given the simplicity of the task, we expected instructed learning to be sufficient to establish cue-outcome associations and in-scanner learning to be minimal. However, to examine whether our results may have been influenced by any learning that took place during early trials, we examined anticipatory activity in runs 2, 3, and 4 only, after participants had had an additional 16 trials of training during run 1. This analysis did not reveal additional anticipatory activity or group differences in cue-related effects in striatum or other regions associated with reward processing. This suggests that although we are unable to assess the extent to which learning may have taken place in the scanner, it is not likely that additional pre-scan training would have resulted in stronger anticipation effects.

Another important limitation of this study is that patients were taking antipsychotic medications that block dopamine receptors, potentially affecting reward-related brain activation. However, our medication analyses failed to reveal significant relationships between antipsychotic dose and anhedonia score or BOLD signal within the regions of interest. These analyses suggest that the results reported here are not likely to be accounted for by medication effects, although studies including unmedicated patients are required to fully appreciate the extent to which medication may influence the processes examined here.

In sum, these findings suggest that while at the group level there were no differences between patients and controls in striatal activity during reward anticipation or receipt, those individuals with schizophrenia who were higher in anhedonia showed decreased striatal and VMPFC activity in anticipation of reward, even in the absence of requirements for response selection and execution. This suggests that the process of reward prediction may be abnormal in those patients experiencing the most severe anhedonia and motivational deficits.

## Supporting Information

Figure S1 **Regions of interest mask used in voxelwise ROI analyses.**
(TIF)

Figure S2 **Caudate, Putamen, and Nucleus Accumbens ROIs used in mean-activation ROI analyses.**
(TIF)

Figure S3 **Receipt-related activation magnitudes for expected and unexpected rewards and nonrewards in left VLPFC (–44,+22, –6). Magnitudes shown represent mean activation across all voxels in the region.**
(TIF)

Figure S4 **Cue-related activation for both cue types combined (money and no-money) across both groups (patients and controls). Color scale represents Z value in one-sample t-test.**
(TIF)

Text S1 **Movement and signal-to-noise ratio in fMRI data.**
(DOC)

Table S1 **Incremental movement and signal-to-noise ratio in patients and controls.**
(DOC)

Table S2 **Post-hoc paired t-tests for unexpected versus expected rewards and non-rewards within patient and control groups.**
(XLS)
