# Supplementary figures and images for: Pavlovian Reward Prediction and Receipt in Schizophrenia: Relationship to Anhedonia
Source: PLoS One. 2012 May 4;7(5):e35622. doi: 10.1371/journal.pone.0035622 (PMC3344823; doi:10.1371/journal.pone.0035622)

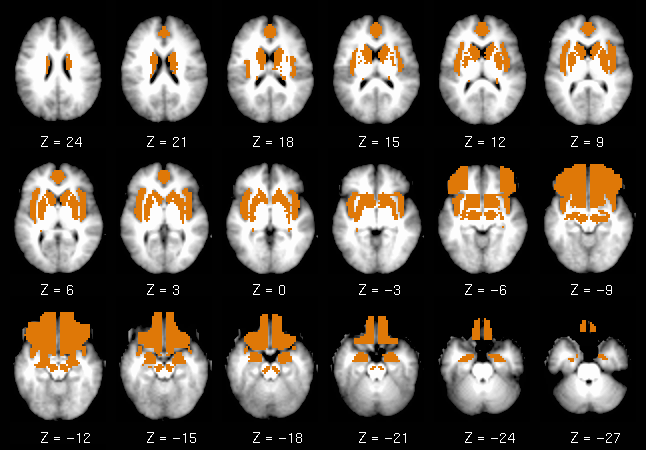

Supplement: Figure S1 — Regions of interest mask used in voxelwise ROI analyses. (TIF) [file pone.0035622.s001.tif]

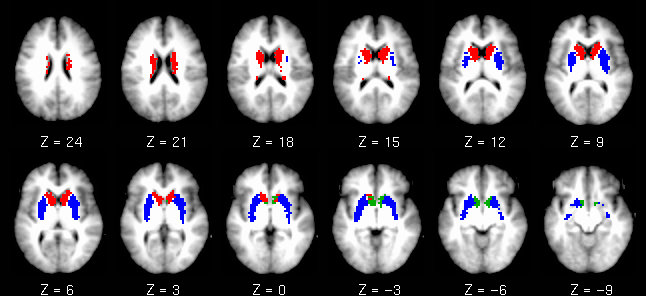

Supplement: Figure S2 — Caudate, Putamen, and Nucleus Accumbens ROIs used in mean-activation ROI analyses. (TIF) [file pone.0035622.s002.tif]

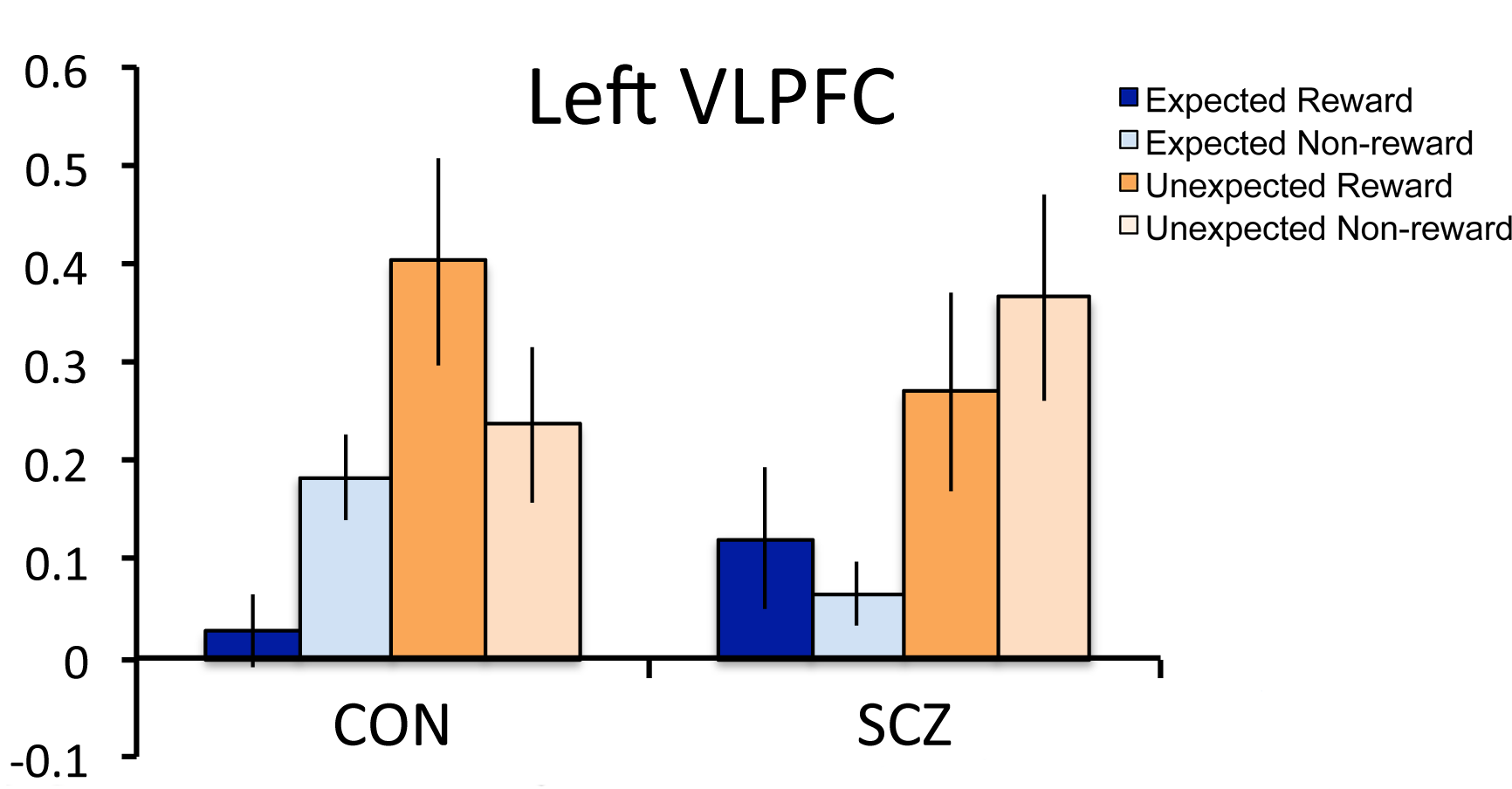

Supplement: Figure S3 — Receipt-related activation magnitudes for expected and unexpected rewards and nonrewards in left VLPFC (–44,+22, –6). Magnitudes shown represent mean activation across all voxels in the region. (TIF) [file pone.0035622.s003.tif]

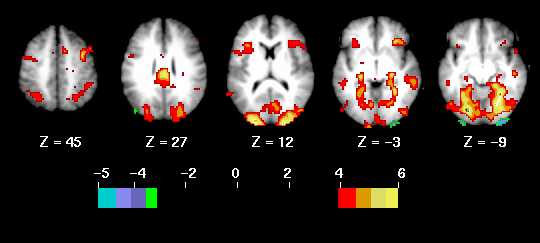

Supplement: Figure S4 — Cue-related activation for both cue types combined (money and no-money) across both groups (patients and controls). Color scale represents Z value in one-sample t-test. (TIF) [file pone.0035622.s004.tif]
