# Supplementary material for: Pavlovian Reward Prediction and Receipt in Schizophrenia: Relationship to Anhedonia
Source: PLoS One. 2012 May 4;7(5):e35622. doi: 10.1371/journal.pone.0035622 (PMC3344823; doi:10.1371/journal.pone.0035622)
Supplement: Text S1 — Movement and signal-to-noise ratio in fMRI data. (DOC) [file pone.0035622.s005.doc]

Text S1

*Movement and Signal-to-Noise Ratio in fMRI Data:* Four patients and two controls were excluded from analysis for excessive movement and/or low signal-to-noise ratio (SNR). Mean incremental (frame-to-frame) movement was computed for each run for each subject and used to compare movement between groups. SNR was computed by determining the ratio of the mean signal intensity to its standard deviation for each frame within each run, and mean SNR values were then calculated for each participant. For the final sample of 25 patients and 20 controls, mean SNR and incremental movement for each translation axis (x, y, z) and rotation axis (pitch, roll, yaw), as well as group t-tests results for each, are summarized in Table S1. The groups differed significantly only in movements along the y axis. Further, neither SNR nor movement along any axis correlated significantly with Chapman physical or social anhedonia score in patients. Together, these results indicate that the groups were well matched for signal quality and that poor signal quality in patients is unlikely to contribute to the group or correlation results reported here.
