# Supplementary material for: Pavlovian Reward Prediction and Receipt in Schizophrenia: Relationship to Anhedonia
Source: PLoS One. 2012 May 4;7(5):e35622. doi: 10.1371/journal.pone.0035622 (PMC3344823; doi:10.1371/journal.pone.0035622)
Supplement: Table S1 — Incremental movement and signal-to-noise ratio in patients and controls. (DOC) [file pone.0035622.s006.doc]

| **Table S1.** Incremental movement and signal-to-noise ratio | | | | | |
| --- | --- | --- | --- | --- | --- |
|  | Control | | Schizophrenia | |  |
|  | Mean | SE | Mean | SE | *p* |
| x | 0.028 | 0.003 | 0.029 | 0.005 | 0.766 |
| y | 0.063 | 0.008 | 0.094 | 0.012 | 0.062 |
| z | 0.063 | 0.008 | 0.085 | 0.013 | 0.182 |
| pitch | 0.055 | 0.006 | 0.067 | 0.008 | 0.243 |
| roll | 0.024 | 0.002 | 0.029 | 0.003 | 0.212 |
| yaw | 0.021 | 0.002 | 0.026 | 0.004 | 0.333 |
| SNR | 276.34 | 72.83 | 277.90 | 76.11 | 0.945 |
| SE, standard error; SNR, Signal-to-noise ratio | | | | | |
